# Clinical Efficacy of In-Line Mechanical Insufflation–Exsufflation in Patients with Invasive Mechanical Ventilation After Cardiopulmonary Bypass

**DOI:** 10.31083/RCM45426

**Published:** 2026-01-16

**Authors:** Dannuo Han, Chenglong Li, Ming Jia, Hong Wang, Liangshan Wang, Xiaotong Hou

**Affiliations:** ^1^Center for Cardiac Intensive Care, Beijing Anzhen Hospital, Capital Medical University, 100029 Beijing, China

**Keywords:** in-line mechanical insufflation–exsufflation, catheter suction, post-cardiopulmonary bypass, invasive mechanical ventilation

## Abstract

**Background:**

This study aimed to evaluate the clinical efficacy of in-line mechanical insufflation–exsufflation (IL-MIE) in airway secretion management in patients receiving invasive mechanical ventilation after cardiopulmonary bypass (CPB).

**Methods:**

A total of 56 patients who underwent CPB and required invasive mechanical ventilation in the Cardiac Surgery Intensive Care Unit of Beijing Anzhen Hospital, Capital Medical University, between July 2015 and July 2020, were enrolled and divided into an IL-MIE group (n = 28) and a conventional suction (CS) group (n = 28). The IL-MIE group received automated secretion clearance every 30 min for 8 h, supplemented with CS as needed, whereas the CS group received standard CS treatment. General patient data, respiratory and hemodynamic parameters, ventilator settings, CS frequency, mechanical ventilation duration, and intensive care unit (ICU) length of stay were recorded during the 8 h intervention.

**Results:**

At 4 h and 8 h, the IL-MIE group exhibited significantly higher arterial oxygen partial pressure, oxygenation index, and static compliance and low plateau pressure (*p* < 0.05). Heart rate was significantly lower in the IL-MIE group at 4 h ((99.21 ± 13.87) vs. (89.32 ± 10.66); *p* < 0.01) and 8 h ((96.71 ± 14.47) vs. (89.61 ± 9.34); *p* = 0.033). The IL-MIE group required fewer CS interventions (0 (0, 1) vs. 4 (3, 4); *p* < 0.01) and had a shorter duration of mechanical ventilation (20 (16.75, 22) vs. 24 (18.75, 26.5); *p* = 0.029) than those in the CS group.

**Conclusions:**

By mimicking physiological airway clearance, IL-MIE significantly improves oxygenation and lung compliance, reduces the duration of mechanical ventilation, and maintains hemodynamic stability during respiratory management in patients after CPB.

## 1. Introduction

Lung injury is a major complication after cardiopulmonary bypass (CPB) [1]. The primary causes of post-CPB pulmonary dysfunction include atelectasis, pulmonary ischemia-reperfusion injury, systemic inflammatory response syndrome, pulmonary microthrombus formation, and transfusion-related acute lung injury [2, 3]. The interruption of mechanical ventilation (MV) during CPB is associated with the development of microatelectasis, hydrostatic pulmonary edema, and surfactant diffusion abnormalities. Consequently, pulmonary function declines. Clinically, this manifests as alveolar collapse, ventilation/perfusion mismatch, reduced lung compliance, and mucus retention, which may further precipitate atelectasis and ventilator-associated pneumonia (VAP).

The inflammatory cascade triggered by CPB leads to pulmonary capillary endothelial injury, facilitating the extravasation of protein-rich exudates into the alveolar and interstitial spaces. Concurrently, ischemia-reperfusion injury impairs ciliary beat frequency and mucociliary transport rate, resulting in increased mucus viscosity and retention in the small airways. This process establishes a vicious cycle of “mucus plugging-alveolar collapse-elevated infection risk”. Therefore, effective airway clearance is essential for improving oxygenation and serves as a core strategy to interrupt the progression of pulmonary complications.

In current clinical practice, conventional suction (CS) is used to clear airway secretions. This technique employs negative pressure to directly remove secretions from the major airways but is associated with several limitations. Catheter insertion can induce hemodynamic fluctuations and cause mechanical trauma to the tracheal mucosa, thereby triggering localized inflammation. Repeated procedures further increase the risk of mucosal bleeding and compromise the epithelial barrier integrity [4, 5], thereby increasing the likelihood of VAP [6, 7]. Notably, CS primarily targets the main bronchi and upper airways and has limited efficacy in clearing deep-seated secretions, resulting in suboptimal clearance. Moreover, manual suctioning requires frequent interruptions of ventilation and manual operation, rendering it highly dependent on nursing procedures. Technical proficiency directly influences therapeutic outcomes, thereby introducing variability into clinical effectiveness.

In-line mechanical insufflation–exsufflation (IL-MIE) employs a mechanical simulation of the airflow dynamics observed during the physiological cough process, specifically the “insufflation–exsufflation” cycle. During the inspiratory phase, positive pressure is applied to ensure optimal alveolar expansion. Subsequently, during the expiratory phase, the system rapidly switches to high negative pressure, generating a high-velocity expiratory airflow. This airflow transfers kinetic energy to secretions, propelling them upward into the tracheobronchial tree and facilitating the mobilization of peripheral secretions toward the proximal airways [8]. This process can be performed without disconnecting the ventilator, thereby ensuring continuous lung-protective ventilation. Recent studies have investigated the therapeutic efficacy of MIE in critically ill patients receiving MV [9, 10, 11]. As a noninvasive modality, MIE demonstrates minimal hemodynamic impact compared with that of CS and is associated with low incidence rates of trauma and VAP [12]. No adverse safety events have been reported with its use [13]. Evidence confirms the significant clinical value of MIE in facilitating weaning in patients within the intensive care unit (ICU) [14], and its application in chronic noninvasive ventilation has been increasingly adopted [15].

During the early postoperative period after CPB, patients frequently exhibit significant hemodynamic fluctuations, necessitating invasive positive-pressure ventilation, stringent monitoring of the oxygenation index (OI), and intensive airway management. This study aimed to evaluate the clinical utility of IL-MIE in patients receiving invasive MV during the early postoperative phase after CPB.

## 2. Methods

### 2.1 Patient and Study Setting 

This historical, single-center, observational study was conducted at Beijing Anzhen Hospital, Capital Medical University, China. Patients aged between 18–75 years who underwent invasive MV after CPB cardiac surgery between July 2015 and July 2020 were enrolled. Written informed consent was obtained from all patients or their legal guardians. Patients were excluded if they had any of the following contraindications: acute spinal shock (within 48 h after spinal cord injury, based on neurological examination records); recent respiratory tract trauma or surgery (within the past 4 weeks, confirmed by surgical records or bronchoscopy reports); conditions including rib fractures, pneumothorax (based on chest computed tomography (CT) imaging reports obtained within 24 h of admission), or hemoptysis (>20 mL within 24 h according to nursing records); patients requiring high positive end-expiratory pressure ventilation (>10 cm H_2_O) because of cardiogenic pulmonary edema (X-ray showing pulmonary edema, echocardiography indicating LVEF <40%, and BNP levels >500 pg/mL), acute respiratory distress syndrome (Berlin Definition), or severe ischemic heart disease (acute myocardial infarction within the past 3 months, LVEF <35%, or left main and/or multivessel coronary stenosis >70%). Patients with incomplete clinical data were excluded. The patients were divided into CS and IL-MIE groups. The CS group received standard catheter suction therapy, whereas the IL-MIE group was treated with the IL-MIE device in addition to conventional therapy. All patients received invasive MV after CPB surgery and basic treatments, including analgesia, sedation, cardiotonics, diuretics, vasodilation, anti-infection therapy, anticoagulation, and nutritional support. This study was approved by the Beijing Anzhen Hospital Human Research Ethics Committee (Ethics number: 2025215x, Beijing Anzhen Hospital), and all procedures operations were performed in accordance with the relevant guidelines and regulations.

### 2.2 Patient Management

In the CS group, patients were administered 100% oxygen for 1 min before CS intervention, according to the ICU’s standard operating protocol. The observation period for the CS group was 8 h. CS was performed whenever patients exhibited symptoms of airway secretion accumulation, such as a sawtooth waveform on the ventilator’s volume–pressure curve, audible rhonchi on auscultation, increased peak airway pressure (volume-controlled ventilation mode), decreased tidal volume (pressure controlled ventilation mode), oxygen saturation below 95%, visible airway secretions in the respiratory circuit, acute respiratory distress, suspected aspiration of gastric contents or upper airway secretions.

**Fig. 1.**
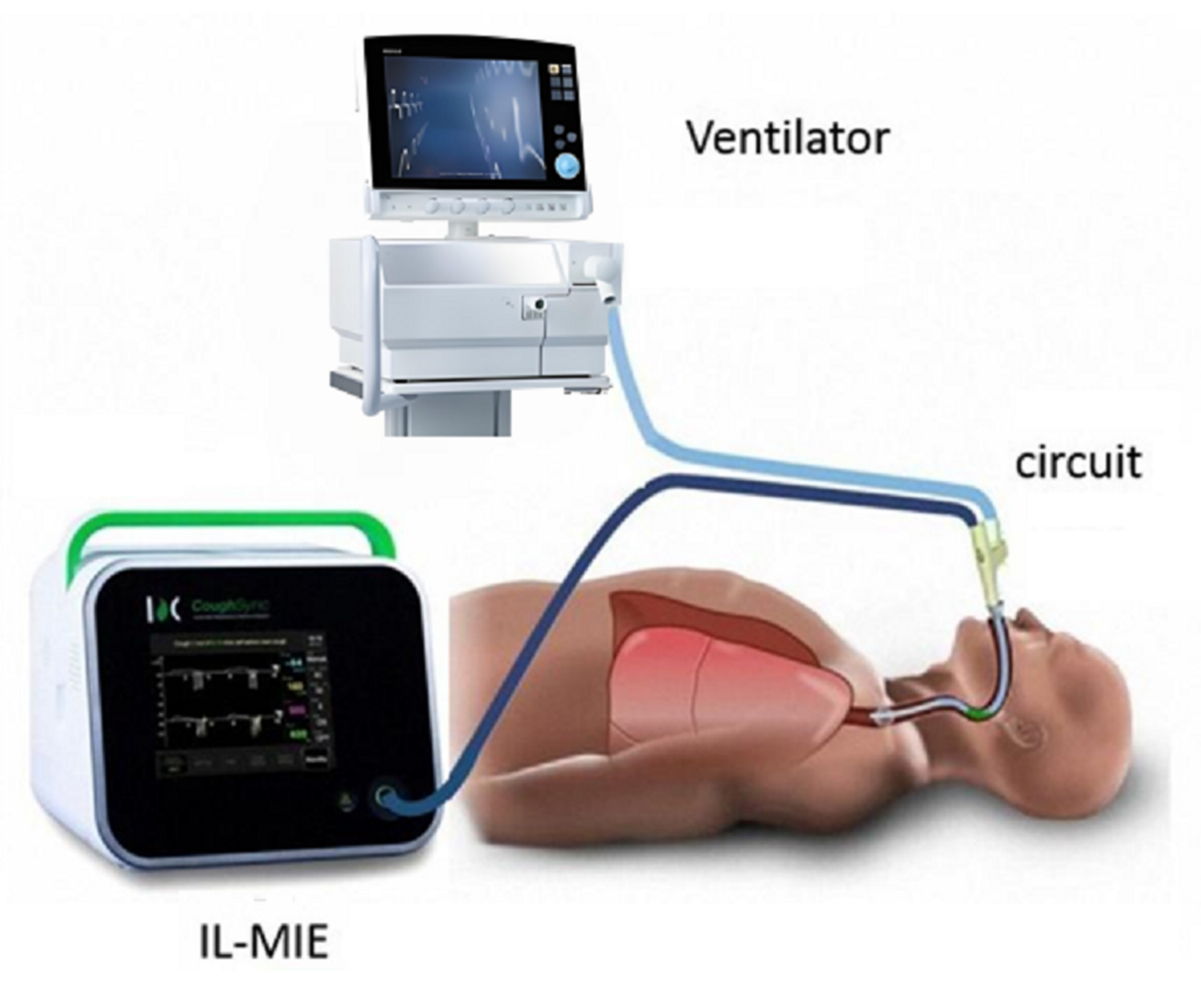
**Setup and functioning of in-line mechanical insufflation–exsufflation (IL-MIE)**.

The IL-MIE device (RC001-01A, CoughSync, Ruxin Technology Development Co., Ltd., Beijing, China) was used for 8 h, with automated secretion clearance performed every 30 min. Supplemental suctioning was performed using criteria identical to those in the CS group. No supplemental oxygen beyond the baseline requirement was provided to the IL-MIE group. The IL-MIE device operated 10 cycles per treatment, with an exsufflation pressure of –60 cmH_2_O. Accumulated airway secretions in the sputum collection cup were cleared regularly (Fig. 1).

### 2.3 Data Collection 

General information and relevant data were collected at 0, 4, and 8 h after treatment initiation. Respiratory-related clinical parameters included arterial oxygen partial pressure, OI, and arterial carbon dioxide partial pressure. The recorded ventilator parameters were tidal volume, peak inspiratory pressure, airway plateau pressure (Pplat), and static compliance (Cst). Circulation-related clinical indicators included heart rate (HR) and mean arterial pressure (MAP). Additionally, the frequency of endotracheal suction, duration of MV, and length of ICU stay were recorded during the observation period.

### 2.4 Statistical Analysis

All analyses were performed using SPSS (version 25.0; IBM Corp., Armonk, NY, USA). Categorical variables and frequencies were presented as percentages, and continuous variables as “Mean ± SD” or “Median (IQR)” according to their distribution. The normality of distribution was tested using the Kolmogorov-Smirnov test. Normally distributed data were compared using Student’s independent *t*-test, whereas non-normally distributed data were compared using the Mann-Whitney U test. Categorical variables were compared using the chi-squared test or Fisher’s exact test, as appropriate. Repeated-measures ANOVA was used to further investigate and compare differences between groups and time points. Statistical significance was set at *p* ≤ 0.05.

## 3. Results

### 3.1 Populations

Fifty-six patients were enrolled in the study and stratified into the CS (n = 28) and IL-MIE groups (n = 28). One patient with severe ischemic heart disease was excluded. Normally distributed continuous data were analyzed using the *t*-test, whereas categorical data were analyzed using the chi-square test, specifically for ventilation modes; Fisher’s exact test was applied based on expected frequencies. The CS group comprised 35.7% men, whereas the IL-MIE group included 42.9% men. No significant differences were observed between the two groups in terms of age, body mass index (BMI), history of smoking or drinking, or comorbidities, including hypertension, diabetes, stroke, and chronic obstructive pulmonary disease. Additionally, no significant differences were observed in initial HR or MAP values. Regarding the type of surgery, valvular, aortic, and congenital heart surgeries accounted for 82.1%, 21.4%, and 14.3%, respectively, in the CS group. There were also no differences in ventilator modes between the two groups (Table 1).

**Table 1.** **Clinical characteristics of the patients [$\bar{x}$ ± s, n (%)]**.

Characteristic		CS group (n = 28)	IL-MIE group (n = 28)	T/χ^2^	*p*-value
Men		10 (35.7)	12 (42.9)	0.299	0.584
Age, years		56.11 ± 9.06	55.71 ± 11.83	0.139	0.890
BMI, kg/m^2^		21.38 ± 2.46	21.78 ± 2.72	–0.567	0.573
Smoking		12 (42.9)	17 (60.7)	1.788	0.181
Drinking		16 (57.1)	14 (50.0)	0.287	0.592
Comorbid conditions					
	Hypertension	15 (53.6)	19 (67.9)	1.198	0.274
	Diabetes	15 (53.6)	16 (57.1)	0.072	0.788
	Stroke	5 (17.9)	2 (7.1)	1.469	0.225
	COPD	3 (10.7)	4 (14.3)	1.163	0.686
Type of surgery					
	Valvular surgery	23 (82.1)	20 (71.4)	0.902	0.342
	Aortic surgery	6 (21.4)	5 (17.9)	0.113	0.737
	Congenital heart surgery	4 (14.3)	5 (17.9)	0.132	0.716
Ventilator mode					
	Volume-controlled	25 (89.3)	27 (96.4)		0.611
	Pressure-controlled	3 (10.7)	1 (3.6)		0.611
HR, bpm		80.57 ± 7.73	78.21 ± 8.44	1.089	0.281
MAP, mmHg		80.79 ± 8.32	81.57 ± 7.20	–0.378	0.707

IL-MIE, in-line mechanical insufflation–exsufflation; CS, conventional suction; BMI, body mass index; COPD, chronic obstructive pulmonary disease; HR, heart rate; MAP, mean arterial pressure.

### 3.2 Indicators of Respiration and Circulation

**Fig. 2.**
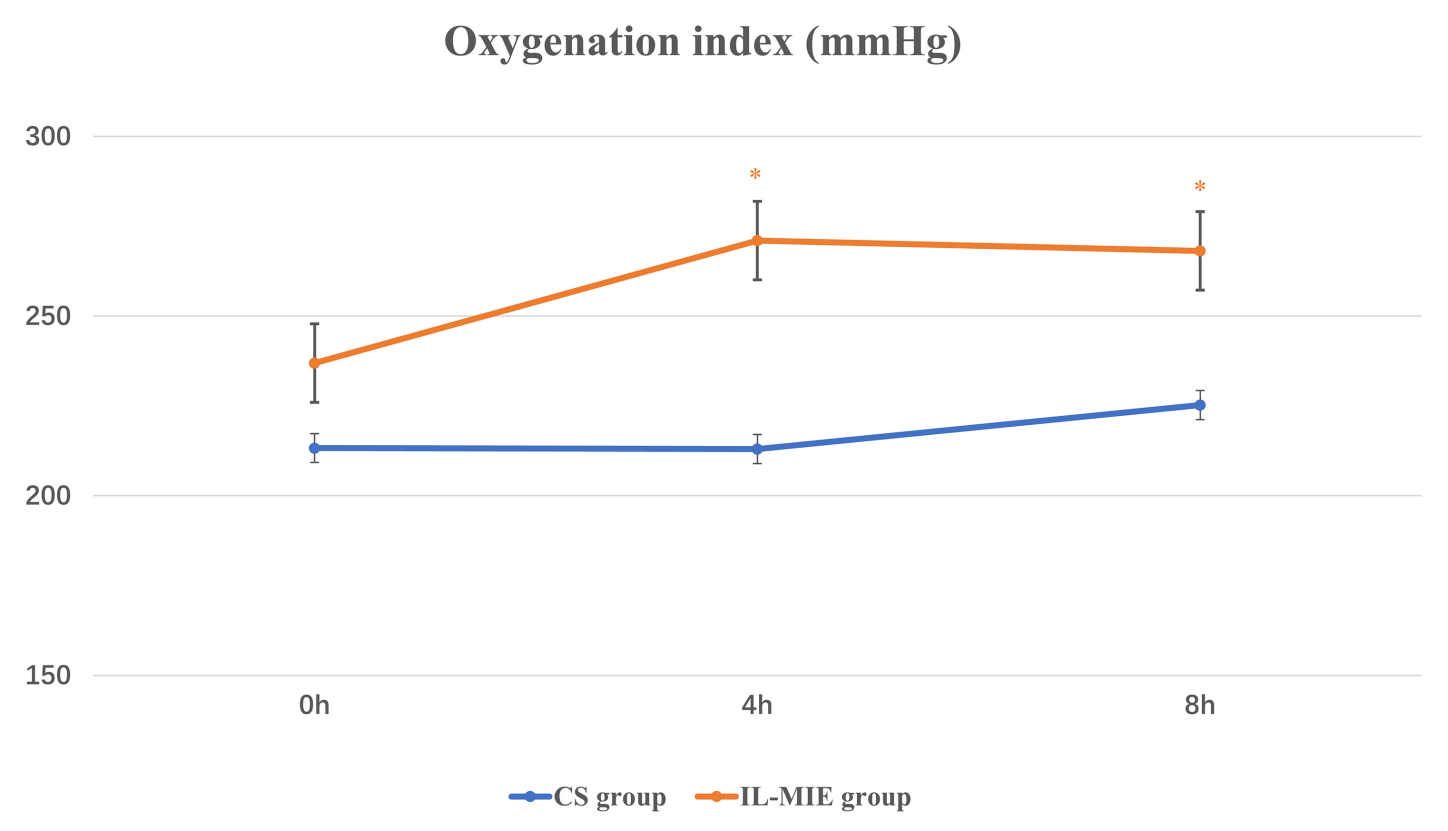
**Oxygenation index**. * *p* ≤ 0.05. IL-MIE, in-line mechanical insufflation–exsufflation; CS, conventional suction.

**Fig. 3.**
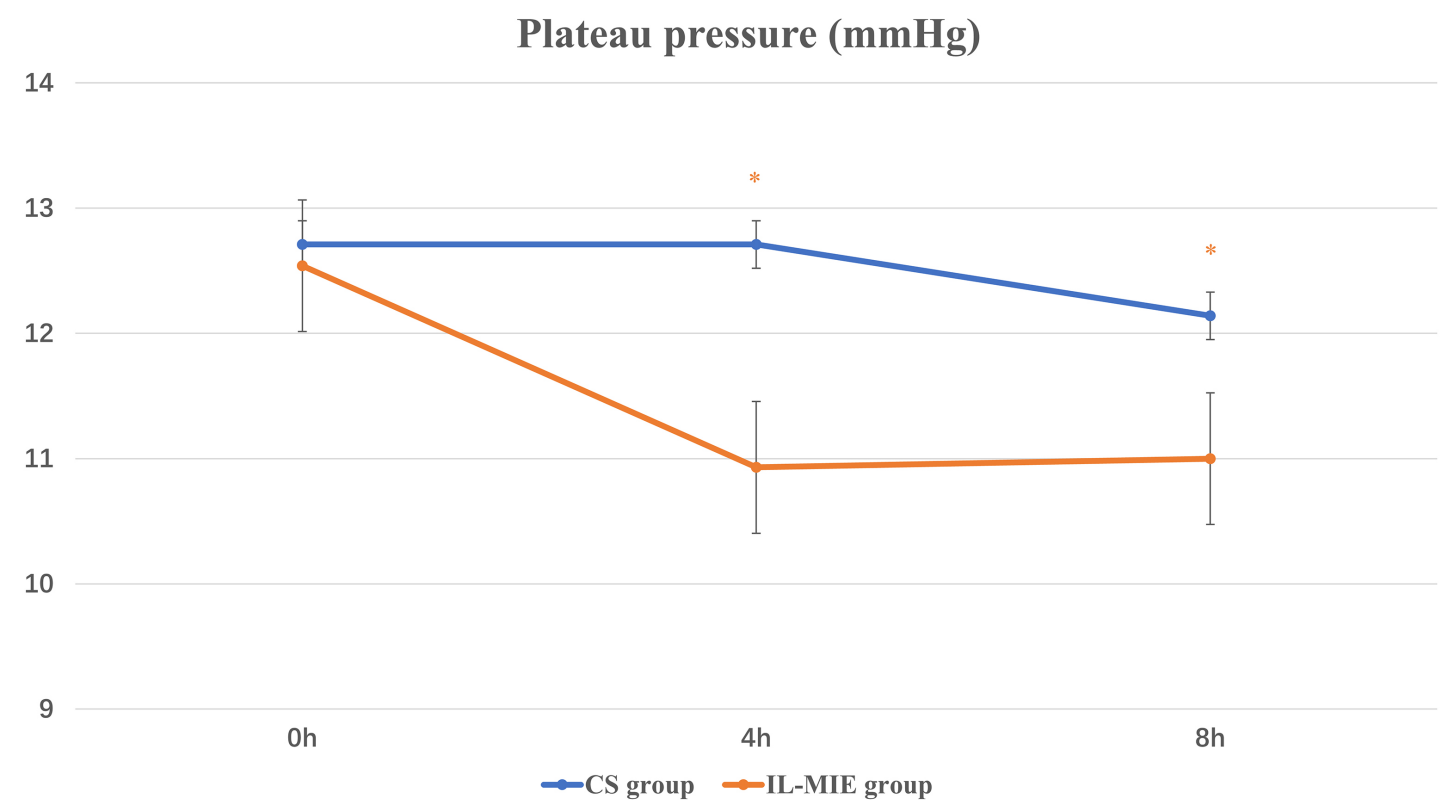
**Plateau pressure**. * *p* ≤ 0.05. IL-MIE, in-line mechanical insufflation–exsufflation; CS, conventional suction.

**Fig. 4.**
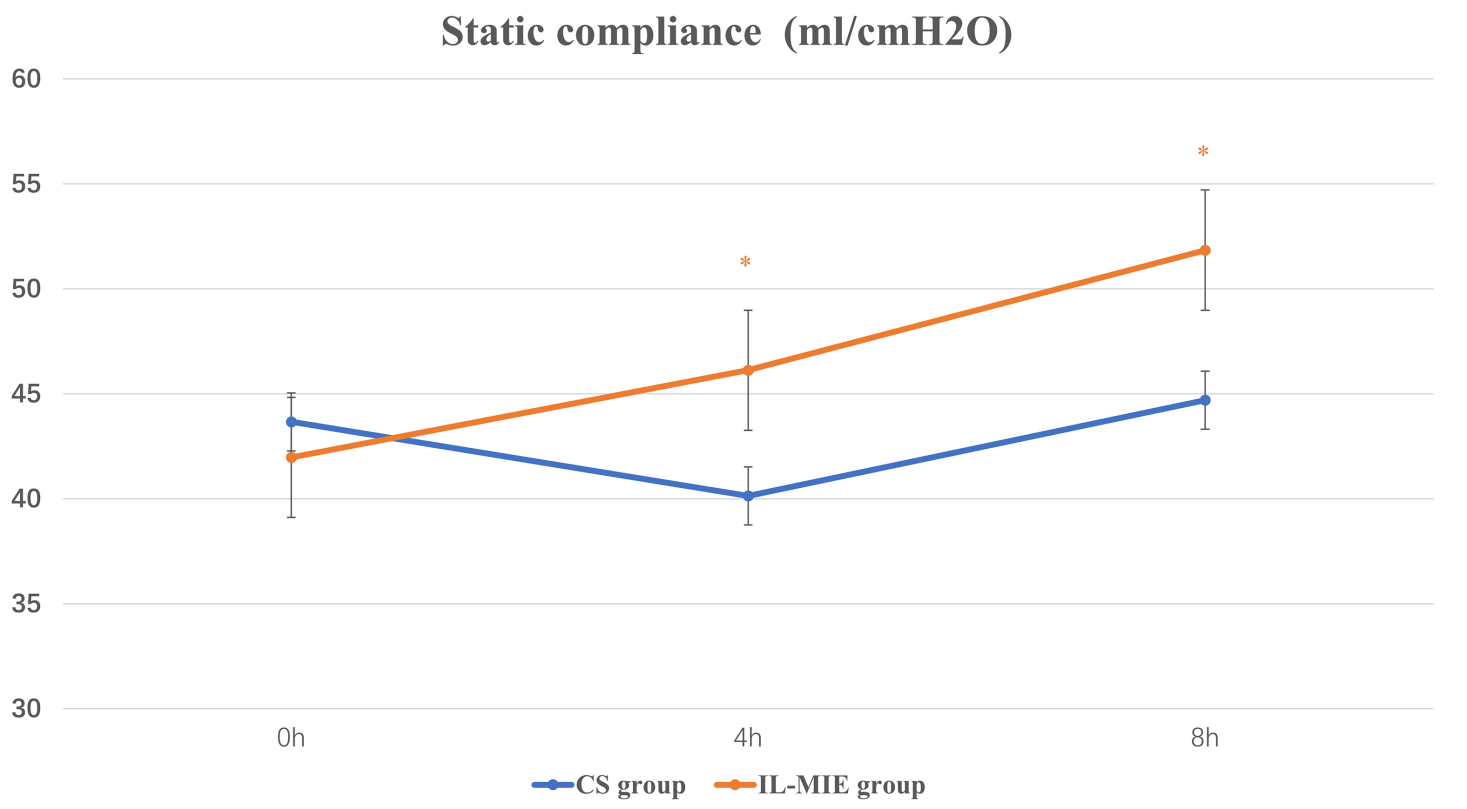
**Static compliance**. * *p* ≤ 0.05. IL-MIE, in-line mechanical insufflation–exsufflation; CS, conventional suction.

**Fig. 5.**
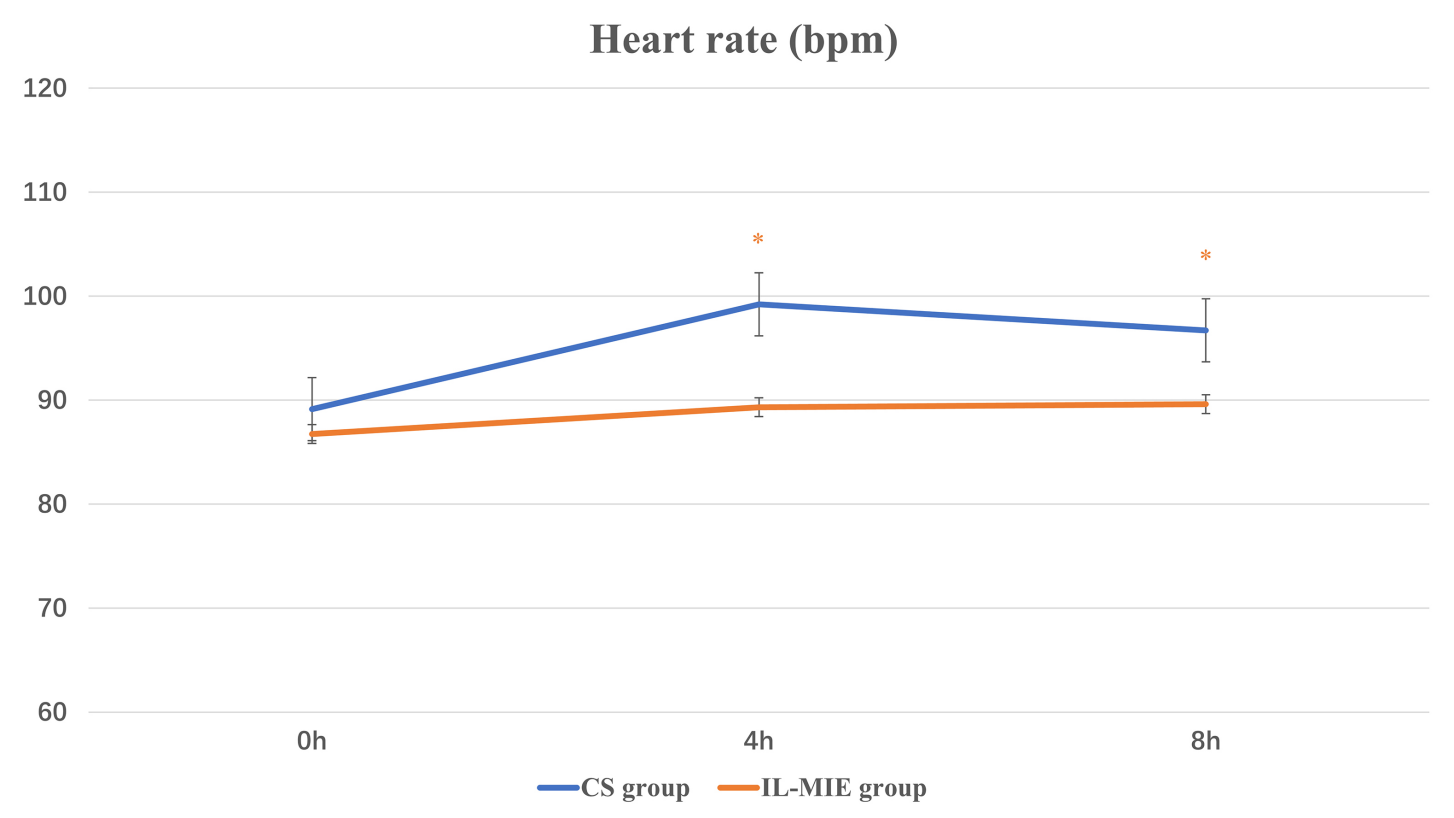
**Heart rate**. * *p* ≤ 0.05. IL-MIE, in-line mechanical insufflation–exsufflation; CS, conventional suction.

Indicators of respiration and circulation were observed at 0, 4, and 8 h (Table 2). A repeated-measures ANOVA was used. At baseline, no significant differences were observed in OI between the two groups. At 4 h, the mean OI value was significantly lower in the CS group than in the IL-MIE group [(212.99 ± 48.30) vs. (270.98 ± 58.20), *p* < 0.01]. Similarly, at 8 h, the mean OI value in the CS group remained low [(225.24 ± 53.93) vs. (268.15 ± 58.21), *p* < 0.01]. A significant interaction effect was observed for OI (*p* = 0.05) (Fig. 2). At 4 h, the mean Pplat value was significantly higher in the CS group than in the IL-MIE group [(12.71 ± 2.12) vs. (10.93 ± 1.63), *p* < 0.01], and he mean Cst value was significantly low [(40.13 ± 7.43) vs. (46.12 ± 12.41), *p* = 0.033]. At 8 h, the mean Pplat value remained high in the CS group [(12.14 ± 1.60) vs. (11.00 ± 1.78), *p* = 0.015], and the mean Cst value remained low [(44.69 ± 6.85) vs. (51.84 ± 13.77), *p* = 0.017]. No differences were observed at 0 h. A significant interaction between time and group was observed for both Pplat and Cst (*p* ≤ 0.05) (Figs. 3,4). Regarding HR, mean values were significantly higher in the CS group than in the IL-MIE group at both 4 h [(99.21 ± 13.87) vs. (89.32 ± 10.66), *p* < 0.01] and 8 h [(96.71 ± 14.47) vs. (89.61 ± 9.34), *p* = 0.033] (Fig. 5). The interaction effect was not significant (*p* = 0.236). No statistically significant differences were observed in arterial carbon dioxide partial pressure, tidal volume, peak inspiratory pressure, or MAP at any time point (*p* > 0.05).

**Table 2.** **Indicators of respiration and circulation [$\bar{x}$ ± s, n (%)]**.

		CS group	IL-MIE group	F-value	*p*-value
PaO_2_, mmHg	0 h	124.07 ± 29.81	136.61 ± 30.43	2.425	0.125
	4 h	112.50 ± 24.32*	129.50 ± 23.78	0.115	0.011
	8 h	108.43 ± 22.28*	132.82 ± 16.44	0.287	0.000
	F-value	6.073	1.773		
	*p*-value	0.004	0.180		
	Main Effect				
	Time (F, P)	5.504, 0.007			
	Group (F, P)	9.363, 0.003			
	Interaction (F, P)	2.341, 0.106			
OI, mmHg	0 h	213.25 ± 56.24	236.90 ± 55.20	2.523	0.118
	4 h	212.99 ± 48.30	270.98 ± 58.20*	16.470	0.000
	8 h	225.24 ± 53.93	268.15 ± 58.21*	8.188	0.006
	F-value	0.845	8.176		
	*p*-value	0.435	0.001		
	Main Effect				
	Time (F, P)	5.386, 0.006			
	Group (F, P)	11.271, 0.001			
	Interaction (F, P)	3.086, 0.050			
PaCO_2_, mmHg	0 h	40.22 ± 5.19	38.84 ± 4.11	1.212	0.276
	4 h	39.82 ± 3.82	38.82 ± 3.06	1.168	0.285
	8 h	39.22 ± 3.58	38.60 ± 2.96	0.495	0.485
	F-value	0.472	0.038		
	*p*-value	0.626	0.963		
	Main Effect				
	Time (F, P)	0.356, 0.701			
	Group (F, P)	3.194, 0.080			
	Interaction (F, P)	0.128, 0.880			
VT, mL	0 h	461.79 ± 61.83	436.79 ± 65.55	2.155	0.148
	4 h	462.50 ± 63.69	442.14 ± 68.17	1.333	0.253
	8 h	463.21 ± 60.68	448.21 ± 67.11*^#^	0.770	0.384
	F-value	0.069	4.465		
	*p*-value	0.943	0.016		
	Main Effect				
	Time (F, P)	2.819, 0.069			
	Group (F, P)	1.389, 0.244			
	Interaction (F, P)	1.715, 0.190			
PIP, cmH_2_O	0 h	19.68 ± 1.98	20.36 ± 1.73	1.866	0.178
	4 h	20.16 ± 2.13	20.07 ± 1.74	0.043	0.837
	8 h	20.07 ± 1.88	20.39 ± 1.89	0.406	0.527
	F-value	0.617	0.221		
	*p*-value	0.543	0.803		
	Main Effect				
	Time (F, P)	0.172, 0.842			
	Group (F, P)	1.124, 0.294			
	Interaction (F, P)	0.579, 0.562			
Pplat, cmH_2_O	0 h	12.71 ± 1.30	12.54 ± 1.29	0.266	0.608
	4 h	12.71 ± 2.12	10.93 ± 1.63*	12.454	0.001
	8 h	12.14 ± 1.60	11.00 ± 1.78*	6.353	0.015
	F-value	0.959	7.934		
	*p*-value	0.390	0.001		
	Main Effect				
	Time (F, P)	5.547, 0.005			
	Group (F, P)	22.264, 0.000			
	Interaction (F, P)	2.994, 0.049			
Cst, mL/cmH_2_O	0 h	43.66 ± 7.49	41.97 ± 7.74	0.689	0.410
	4 h	40.13 ± 7.43	46.12 ± 12.41	4.794	0.033
	8 h	44.69 ± 6.85	51.84 ± 13.77*	6.060	0.017
	F-value	2.353	9.115		
	*p*-value	0.105	0.000		
	Main Effect				
	Time (F, P)	7.628, 0.001			
	Group (F, P)	4.263, 0.044			
	Interaction (F, P)	4.705, 0.011			
HR, bpm	0 h	89.14 ± 10.28	86.75 ± 13.30	0.567	0.455
	4 h	99.21 ± 13.87*	89.32 ± 10.66	8.957	0.004
	8 h	96.71 ± 14.47	89.61 ± 9.34	4.766	0.033
	F-value	4.783	0.453		
	*p*-value	0.012	0.683		
	Main Effect				
	Time (F, P)	4.640, 0.012			
	Group (F, P)	10.469, 0.002			
	Interaction (F, P)	1.463, 0.236			
MAP, mmHg	0 h	77.11 ± 6.40	79.29 ± 5.92	1.748	0.192
	4 h	76.71 ± 5.02	77.89 ± 7.02	0.522	0.473
	8 h	76.57 ± 5.29	74.54 ± 6.10*	1.778	0.188
	F-value	0.060	5.149		
	*p*-value	0.942	0.009		
	Main Effect				
	Time (F, P)	2.895, 0.06			
	Group (F, P)	0.215, 0.645			
	Interaction (F, P)	1.942, 0.148			

PaO_2_, arterial oxygen partial pressure; OI, oxygenation index; PaCO_2_, arterial carbon dioxide partial pressure; VT, tidal volume; PIP, peak inspiratory pressure; Pplat, airway plateau pressure; Cst, static compliance; HR, heart rate; MAP, mean arterial pressure; IL-MIE, in-line mechanical insufflation–exsufflation; CS, conventional suction. * *p* < 0.05 compared with 0 h, # *p* < 0.05 compared with 4 h.

### 3.3 Patient Outcomes

**Fig. 6.**
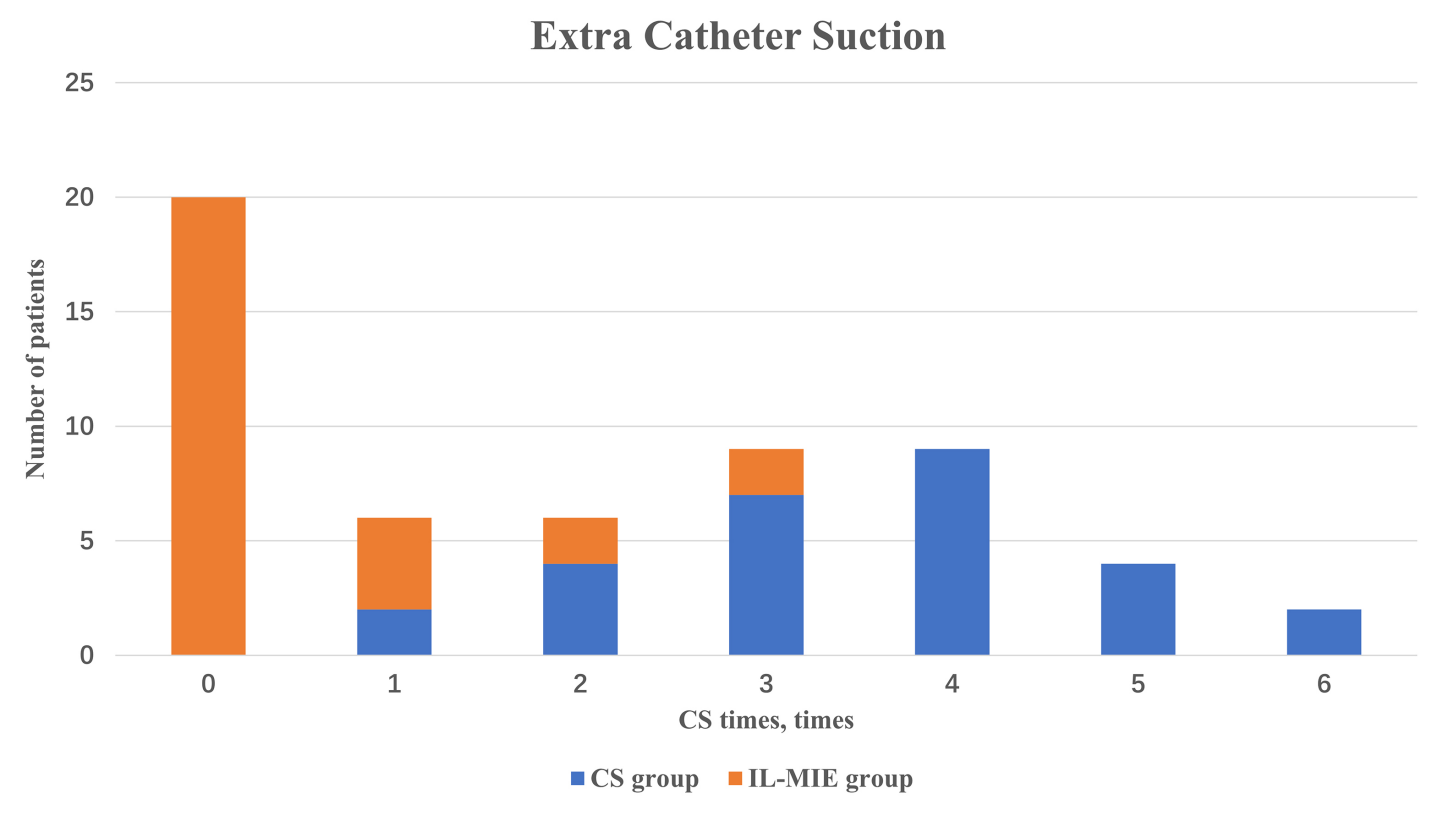
**Extra catheter suction**. IL-MIE, in-line mechanical insufflation–exsufflation; CS, conventional suction.

In the IL-MIE group, the time to additional CS treatment was significantly shorter than in the CS group [0 (0, 1) vs. 4 (3, 4), *p* < 0.01]. In the IL-MIE group, 71.4% (20/28) of patients completely replaced CS therapy with IL-MIE, 14.3% (4/28) required one CS treatment, and 7.1% (2/28) required two or three CS treatments (Fig. 6). The duration of MV was significantly shorter in the IL-MIE group than in the CS group [20 (16.75, 22) vs. 24 (18.75, 26.5), *p* = 0.029]. No statistically significant difference was observed in ICU stay between the groups [22 (20.75, 25.25) vs. 26.5 (20.75, 33), *p* = 0.113] (Table 3). There were no adverse reactions or complications, including severe arrhythmias, hypotension, airway damage, pneumothorax, or bleeding in either group.

**Table 3.** **Patient outcomes [$\bar{x}$ ± s, M (P25, P75)]**.

	CS group (n = 28)	IL-MIE group (n = 28)	U	*p*-value
Extra CS, times	4 (3, 4)	0 (0, 1)	32.000	0.000
MV time, hours	24 (18.75, 26.5)	20 (16.75, 22)	260.000	0.029
VAP, %	2 (7.14)	0 (0)		0.493
ICU stay, hours	26.5 (20.75, 33)	22 (20.75, 25.25)	295.500	0.113

MV, mechanical ventilation; VAP, ventilator-associated pneumonia; IL-MIE, in-line mechanical insufflation–exsufflation; CS, conventional suction; ICU, intensive care unit.

An adjusted analysis for age, type of surgery, and chronic obstructive pulmonary disease status was conducted with respect to OI, duration of MV, and ICU length of stay. No statistically significant differences were observed in any subgroup (Table 4).

**Table 4.** **Patient outcomes in IL-MIE subgroup analysis [$\bar{x}$ ± s, M (P25, P75)]**.

		OI (8 h), mmHg	MV time, hours	ICU stay, hours
Years				
	<60	256.67 (221.67, 306.25)	20.00 (17.00, 26.00)	22.00 (20.50, 27.00)
	≥60	272.50 (220.00, 330.00)	20.00 (16.00, 20.00)	22.00 (20.00, 26.00)
	*p*-value	0.447	0.371	0.763
Types of surgery				
	Valvular surgery	272.72 (239.75, 315.13)	19.00 (16.25, 20.00)	22.00 (20.25, 24.75)
	Aortic surgery	205.00 (183.33, 264.17)	24.00 (18.00, 32.00)	25.00 (19.50, 34.50)
	Congenital heart surgery	273.33 (223.00, 335.30)	20.00 (16.00, 23.00)	24.00 (18.00, 39.50)
	*p*-value	0.113	0.253	0.662
COPD				
	Yes	301.67 (210.83, 348.75)	17.00 (16.00, 19.50)	20.50 (16.25, 38.25)
	No	260.34 (224.58, 306.88)	20.00 (17.00, 22.00)	23.00 (21.00, 25.75)
	*p*-value	0.431	0.131	0.356

OI, oxygenation index; MV, mechanical ventilation; COPD, chronic obstructive pulmonary disease; IL-MIE, in-line mechanical insufflation–exsufflation; CS, conventional suction.

## 4. Discussion

The primary objectives of airway management in mechanically ventilated patients are to maintain airway patency, ensure adequate ventilation, and prevent pulmonary complications. Improper timing, duration, or technique of CS may lead to complications, such as airway mucosal injury, atelectasis, bronchospasm, hypoxemia, infection, hemodynamic instability, arrhythmias, and artificial airway obstruction. In ICU patients with excessive secretions, the frequent use of rigid suction catheters inevitably causes mucosal trauma, induces severe coughing, and exacerbates tissue hypoxia. In patients who underwent cardiac surgery, this may result in alveolar collapse, atelectasis, or hemodynamic fluctuations, thus failing to achieve an optimal balance between safety and efficacy. A meta-analysis by Jongerden *et al*. [16] encompassing 15 studies demonstrated that CS increased bacterial colonization in the airway and elevated the risk of VAP. Therefore, overcoming the limitations of rigid suction catheters, avoiding the interruption of the inspiratory phase during MV, minimizing hemodynamic instability, and improving weaning rates are critical for effective airway clearance in the ICU.

In this study, OI values were significantly higher in the IL-MIE group than in the CS group, and the duration of MV was markedly shorter. Notably, the CS group required 100% oxygen supplementation during each suction procedure, whereas the IL-MIE group did not require supplemental oxygen before or after treatment. Moreover, over the 8 h treatment period, OI values in the IL-MIE group were significantly improved compared with those in the CS group. Inadequate secretion clearance is a major risk factor for failed ventilator weaning in ICU patients [17, 18, 19]. CS often fails to reach the left main bronchus [20] and is less effective in clearing secretions beyond the first-generation bronchi. By contrast, physiological coughing can effectively clear secretions from the 7th–8th generation small bronchi [21], with comparable efficacy in both the left and right airways [22]. This suggests that IL-MIE may outperform CS in clearing secretions from the left lung and deeper small airways beyond the first-generation bronchi. Although OI represents indirect evidence, it serves as a key clinical indicator of improved pulmonary ventilation and gas exchange resulting from effective clearance of deep secretions.

The IL-MIE group exhibited significantly lower Pplat and higher lung compliance than the CS group. Reduced lung compliance decreases the effective tidal volume per unit time, impairing oxygenation and tissue perfusion, thereby adversely affecting the efficacy of MV. For instance, in patients with severe acute respiratory distress syndrome and poor alveolar compliance, alveolar volume often fails to normalize within a standard respiratory cycle post-suction, predisposing them to atelectasis. However, reducing suctioning frequency or duration may lead to inadequate secretion clearance, increased VAP incidence, and prolonged invasive ventilation. When IL-MIE is performed in a mode where the exsufflation volume does not exceed the patient’s tidal volume, it minimizes alveolar collapse, preserves lung compliance, and shortens the duration of invasive ventilation. A significant improvement in Cst was observed in the IL-MIE group after intervention. Increased compliance indirectly reflects alveolar recruitment and improved airway patency, which is consistent with the effects of secretion clearance.

There were no significant differences in VAP rates or ICU stay between the two groups. The development of VAP is a multifactorial process. The key target of IL-MIE is secretion accumulation and aspiration. Biofilm formation, oral bacterial colonization, and a decline in host immunity are also contributing factors to VAP. Similarly, many factors influence ICU stay, including multi-organ function, infection, and nutritional status. However, it is difficult to determine the impact of IL-MIE on long-term outcomes. In our study, the determination of both the 8 h treatment duration and 30 min intervention interval for IL-MIE was based on considerations of hemodynamic stability and clinical workflow. Future research could employ a randomized design to investigate whether increasing frequency or extending overall treatment duration might further reduce the need for CS, improve oxygenation, or affect long-term outcomes. Our study provides a foundational framework and reference for exploring this critical parameter, and determining the optimal regimen should be the key focus of subsequent investigations.

Our findings indicate that IL-MIE provides a safe and stable continuous, timed, and automated airway clearance therapy. Compared with the CS group, no significant increase in HR was observed in the IL-MIE group. Moreover, during the observation period, mean HR values were lower in the IL-MIE group than in the CS group at both 4 h and 8 h. However, MAP showed no significant intergroup differences. This suggests that noninvasive secretion management with IL-MIE promotes hemodynamic stability. IL-MIE is particularly valuable for maintaining hemodynamic stability in patients after cardiac surgery. IL-MIE significantly reduced or eliminated the need for CS in mechanically ventilated patients. Specifically, 71.4% of IL-MIE-treated patients did not require CS, whereas the remaining 21.4% experienced a >50% reduction in CS frequency. In patients with a high secretion burden, IL-MIE may substantially decrease ICU workload.

We conducted a secondary analysis of specific indicators in the IL-MIE group based on surgery type, age, and underlying pulmonary disease. However, no statistically significant differences were observed in the subgroup analysis. Future prospective studies with larger sample sizes or longer treatment durations may help minimize bias and validate these findings.

This study had several limitations. First, it was a single-center, historical, non-randomized controlled trial, which may introduce potential bias. The small sample size may have affected the generalizability of our results. Multicenter, randomized controlled trials with larger cohorts are needed to validate these findings. Second, the observation period was limited to 8 h, whereas post-cardiac surgery patients often require prolonged MV. In future studies, we will extend the observation period or increase follow-up data to evaluate potential long-term clinical benefits. Third, a direct assessment of secretion clearance efficacy via dry weight measurements was not performed. Cst reflects alveolar recruitment and improved airway patency, which align with the effects of secretion clearance. However, the ultimate goal of airway clearance is to improve oxygenation and ventilation efficiency and reduce ventilation duration, rather than merely removing secretions of a specific weight.

## 5. Conclusions

The IL-MIE achieves secretion clearance by closely mimicking physiological airway clearance mechanisms. It significantly improves oxygenation and lung compliance, shortens the duration of MV, and maintains hemodynamic stability during the respiratory management of patients after CPB. Its automated, continuous operation reduces clinical workload. Future multicenter, large-scale randomized controlled trials are necessary to further validate the efficacy of this technology.

## Data Availability

All data generated during this study are included in this article. The datasets used and analyzed during the current study are available on reasonable request.

## Abbreviations

CPB, cardiopulmonary bypass; MV, mechanical ventilation; VAP, ventilator-associated pneumonia; CS, conventional suction; IL-MIE, in-line mechanical insufflation–exsufflation; OI, oxygenation index; Pplat, airway plateau pressure; Cst, static compliance; HR, heart rate; MAP, mean arterial pressure.
